# Game-Based Learning in Pharmacy Education

**DOI:** 10.3390/pharmacy10010011

**Published:** 2022-01-06

**Authors:** Julie H. Oestreich, Jason W. Guy

**Affiliations:** 1 Department of Pharmaceutical Sciences, College of Pharmacy, University of Findlay, Findlay, OH 45840, USA; julie.oestreich@findlay.edu; 2 Department of Pharmacy Practice, College of Pharmacy, University of Findlay, Findlay, OH 45840, USA

**Keywords:** game-based learning, pharmacy, educational games, serious games, technology, pharmacy education, pharmacy assessment

## Abstract

Game-based learning (GBL) involves adding game elements to non-game activities to encourage engagement. Pharmacy curricula are required to incorporate active learning to meet accreditation standards. The literature supports that well-designed GBL holds the attention of students and improves knowledge in some instances. Furthermore, these adaptable experiences can be leveraged for a variety of content areas in pharmacy education. Some activities utilized by educators require large amounts of technological expertise, while others involve minimal use of technology. The incorporation of technology can create highly immersive experiences for learners; however, there are barriers (e.g., financial and technology prowess) to implementation compared to simpler designs. One area of GBL that is not well defined in the literature is how to adequately assess student learning outcomes. Most current studies describe subjective attitudes and confidence or assess content knowledge through objective pre- and post-tests. In the future, more defined and connected methods for assessment—such as active demonstrations within the game—will be needed to better incorporate GBL into pharmacy curricula. Based on the collective evidence in the literature, some GBL activities may serve as useful tools to improve pharmacy student engagement and learning.

## 1. Introduction

Game-based learning (GBL) applies typical elements of game playing to other non-game areas of activity to encourage engagement [1]. Other terms that have been used in the literature to describe this process include “educational games”, “serious games”, and “gamification” [2]. Examples of GBL activities described in the literature include escape rooms, software and real-life simulations, and quiz-based review games. GBL has been used as a form of active learning in education to help immerse students with material and to prevent inattention during long class sessions. Other reasons to consider GBL include the ability to provide (1) low-stakes formative assessments to students and (2) instant and incremental feedback and results. Some research suggests that integrating low-stakes formative assessments helps increase student achievement [3], as these types of assessments often include short and direct quizzes or assignments that represent a small percentage of the course grade. This strategy can be especially useful when students are provided with instantaneous feedback inside of an immersive game experience.

While GBL is used in a variety of disciplines, one specific area that has grown significantly in the literature is in the education of pharmacists. Active learning is now a required component of the Accreditation Council for Pharmacy Education (ACPE) [4]. In addition, current accreditation standards require colleges of pharmacy to assess student educational outcomes through formative and summative assessments and demonstrate readiness for practice [4]. Although pharmacy curricula differ from institution to institution, all US affiliated institutions must meet ACPE standards; numerous other countries must follow similar guidelines for content. Based on these accreditation standards, pharmacy educators have developed novel approaches to engage learners and supplement didactic teaching methods with active learning. One immersive way to engage learners through active learning and meet ACPE standards is through the utilization of GBL activities.

The content covered in a pharmacy curriculum is varied and includes many different scientific fields and therapeutic areas related to pharmacy. Colleges may find it challenging to incorporate effective learning methods across the curriculum because topics are so diverse and complicated. Therefore, the advantages of GBL as a flexible and adaptable learning tool may prove useful for training students across a variety of topic areas.

GBL can range from simple with no use of technology to many forms and levels of technological integration within the gaming activity. The amount of technology used generally depends on the objective and purpose of the activity. Some of the most sophisticated games include software development to produce real-life simulations of pharmacy practice and content, while other GBL experiences do not rely on technology at all. GBL of all levels of technology can effectively engage students and enhance learning outcomes.

This review will highlight current evidence related to GBL content areas, design, and assessment.

## 2. Methods

A review of the literature was conducted utilizing PubMed and the search terms “game-based learning AND pharmacy”, “serious games AND pharmacy”, “gamification AND pharmacy”, “assessment AND game-based learning AND pharmacy”, “assessment AND serious games AND pharmacy”, “assessment AND gamification AND pharmacy”. The searches yielded 84 article results. Inclusion criteria required the article to be focused on a GBL activity related to pharmacy content in the context of student education. Exclusion criteria included GBL activities exclusively for use in a patient population or other health care disciplines. Abstracts were reviewed for relevance and duplicate studies were removed. A total of 31 studies from the primary literature met the inclusion and exclusion criteria. One potential limitation is that poster presentations are not included in the review.

## 3. Content Focus of Game-Based Learning Activities in Pharmacy

Pharmacists are trained in a variety of different topics from therapeutics to management of a pharmacy. ACPE, the North American Pharmacist Licensure Examination (NAPLEX), and other entities provide specific guidance on topics for which pharmacists need to develop competence for licensure [4,5]. Curricula in pharmacy schools cover these topics to help improve student outcomes [6,7,8,9].

GBL can be designed for a variety of topics in pharmacy school curricula to enhance engagement for students while meeting accreditation requirements. The option to create a GBL activity in almost any area or discipline of pharmacy is one advantage to utilizing this pedagogical approach. Furthermore, many games or activities designed for one topic can be altered to address other content areas without starting with a new idea. Of course, one important shortcoming for this approach is gaming fatigue for the learner, which can occur if too many similar activities occur throughout training.

GBL can be an especially effective method to teach students when content is application-based or considered uninteresting by students. Several of the examples described in the literature introduce students to management concepts or the overall processes and logistics of operating a pharmacy. Gaming activities for these areas allow students to practice with a more hands-on approach to apply concepts that may otherwise be difficult to simulate during a traditional lecture. Table 1 provides a list of topics covered in a review of the GBL literature.

Other gaming activities discussed in the literature serve the purpose of reviewing previous content covered in the classroom by other teaching methods. This strategy provides an engaging alternative to increase confidence with previously covered topics as opposed to introducing new content through GBL. Review activities may allow for easier uptake by students because they already have a foundational level of knowledge before playing the game. Even when designed as a review activity, many examples incorporated tutorials to help students navigate the logistics of the game while applying their foundational knowledge on the topic of interest.

In many cases, these innovative GBL activities improved student engagement and confidence with the content, which may provide value when students leave the classroom setting for their clinical experiences. Since students have already practiced in a simulated environment, they potentially may be more prepared to apply their skills in a real-world setting.

## 4. Design of Games in Pharmacy Education

### 4.1. Overview of Technology Incorporated into Game Design

GBL differs in the amount of technology used to deliver the learning exercise. Some GBL activities use sophisticated technology that requires assistance from a third-party developer, while other games involve minimal use of technology.

The use of technology can create more realistic situations using immersive experiences. It can also help automate assessment of students when using GBL for grading purposes in a course. Many of the games developed in the literature have some scoring associated with them, usually for a grade in the course, while others do not, depending on the game. The incorporation of technology often automates the scoring process and provides instant feedback for the user. This timely feedback allows students an opportunity to review the correct answer or successfully complete a scenario before moving on to the next question or level. These types of games offer low-stakes assignments to enhance formative learning in preparation for more summative assessments. For example, Dell et al., Devraj et al., and Khalafalla et al. utilized a timed quiz format for students to practice answering questions related to pharmacotherapy concepts or pharmacy management concepts [10,11,12]. These games incorporated automated scoring and instant feedback. Importantly, some of these activities demonstrated improved scores or engaged students with content [11,12].

Several GBL activities leveraged enhanced technology to create promising 3D models of community pharmacy practice or other simulations of related professional settings [13,14,15,16]. For instance, Bindoff et al. collaborated with a third-party developer to create a digital pharmacy environment with a front desk, shopping area, dispensing computer, and telephones [14]. The simulations are first person from the perspective of a pharmacist and require the user to interact with patients in the game. Students use a laptop or tablet device to move around the simulated environment and interact with patients based on scenarios written by the instructor. The student then completes a medication history and provides recommendations to the patient through dialog options on the screen [14]. Overall, these games led to mixed success in regards to student engagement and enjoyment from the game and resulted in little impact on knowledge scores, although these assessments were restricted to objective exam-style questions [14,15]. In some simulations, students did report increased confidence in their abilities [15]. Additional technology-enhanced games focused on creating player avatars that participants could utilize to interact with game elements to practice certain skills and advance or level up [17,18]. These simulations focused more on the enjoyment and experience of the user as opposed to evaluating impact on formal student outcomes [17,18].

Despite its benefits, a high level of technology in GBL can create some challenges. Games with sophisticated technology often require advanced knowledge of game development to incorporate the activity into the classroom. In some cases, the use of a third party is needed to help create the game or app. As expected, this expertise comes with a cost and creates a barrier to entry for many educators. Enhanced technology also requires the educator to be familiar with the platform or other technological aspects of the game itself. This can add another barrier for instructors, depending on their own familiarity and comfort level with various technologies. An alternative game design with limited use of technology could be beneficial for these instructors.

Indeed, GBL with simpler game designs or low usage of technology can still be engaging and impact student outcomes. Several GBL activities in the literature detail escape rooms, Jeopardy^®^-like, and challenge events (e.g., scavenger hunt, Amazing Race^®^) that incorporate limited technology. For example, six escape rooms [19,20,21,22,23,24] provided a relatively low-tech way of engaging pharmacy students through an interactive activity. The escape rooms also produced some success in learning, as several of the studies showed improved knowledge scores of participants on standard pre- and post-tests [19,20,21,22]. These activities typically possess less start-up expenses than games with high usage of game development technology, often from a third party. Additionally, low-tech games may be easier to implement in some cases and reduce the number of technical glitches. On the other hand, these designs remove the automation associated with high technology use, which can be a potential drawback. In addition, escape rooms and other games with complex interactions may still require significant development effort in addition to instructor time and support for implementation. In one case, effort was estimated at 40 hours [25].

Table 2 provides a list of GBL activities from the literature and their associated use of technology. Many of these activities supplemented foundational material previously covered in the course, although some occurred in lieu of regular course time on the material.

### 4.2. Game-Based Immersive Experiences

GBL varies in the type of experiences created for student learners. Some games build highly immersive experiences through advanced software programs or other sophisticated methods to simulate real-life situations. To increase playability, games may incorporate themes or compelling stories to capture and hold the attention of the user. Simpler approaches without these thematic design features typically result in less immersive experiences. Examples of immersive games are detailed in the literature.

For instance, Ee et al. describe an advanced software program that allows players to manage a city that produces multiple herbal products [26]. Bindoff et al. and Berger et al. both utilized games that provide a 3D simulation of working at a community pharmacy [14,15]. These advanced software designs add complex graphics and a user interface to create highly immersive experiences that simulate real-life situations. The immersive nature of these games improves confidence of students, likely due to the relevance of reproducing clinical settings encountered by students. However, the impact on clinical reasoning and other multifaceted abilities remains unknown, as most reported outcomes do not match the level of complexity of the experience. In fact, the majority of studies only measured clinical knowledge through straightforward objective questions, which, in some cases, did not improve significantly.

Immersive experiences do not have to rely on technology. For example, Korenoski et al. developed an innovative escape room utilizing pre-set locks, clues, and boxes to teach clinical concepts of toxicology [34]. Berthod et al. created an immersive clean room experience with different zones to simulate and train students on chemotherapy good manufacturing processes [19]. These activities did not rely on technology to immerse participants, but instead recreated the setting or applied hands-on activities to produce an in-depth atmosphere. These types of low-tech immersive experiences also increased scores on post-tests and surveys.

More direct and less immersive GBL can also be effective. Khalafalla et al. utilized general group-based quizzing followed by Kahoot!^®^ competition to prepare students for immunology assessments [12]. Nabhani et al. attempted a similar strategy of quizzing students within an online software platform [32]. Cusick et al. reviewed immunology concepts with a Jeopardy^®^-style game using clicker questions [36]. All of these gaming activities focused on a simpler, direct approach of quizzing students with practice questions related to the topic. Two of these strategies led to increased quiz scores [12,32], while some students found this type of learning engaging [36].

## 5. Assessment of Game-Based Learning

Assessment of GBL varies considerably and has not been clearly defined in the literature. Many of the reported activities in pharmacy education focus specifically on the engagement level of students or knowledge score changes from pre-test to post-test, often assessed as multiple-choice or similar questions. In many cases, these objective assessments do not capture assessment of the loftier goals of GBL activities to manage complex clinical scenarios, enhance communication, and apply critical thinking. Table 3 summarizes current GBL activities and their design and assessment.

The purpose of game-based learning is to engage students while meeting educational goals [38]. Ideally, effective GBL activities should be able to show that learning has taken place during or after the game is complete [38]. This remains difficult for instructors, as the assessment of educational outcomes has not been clearly defined in the literature. Moreover, many assessments for GBL focus on intention, attitude, enjoyment, or usefulness [39], although none of these foci measure the impact on educational outcomes. Some GBL activities assess competency development rather than short-term knowledge gain, although these examples are limited. Higher order skills like problem solving are difficult to measure and could require extensive longitudinal studies to document benefit. Additionally, short-term measures such as post-activity knowledge recall are unlikely to serve as useful surrogates for more complex and critical goals of GBL. Based on these challenges, it is not surprising that the literature does not define a best method for assessing GBL. Graafland et al. have described a potential framework of assessing games through description, rationale, functionality, validity, and data protection [40]. However, this format has not been well utilized.

Debate remains whether GBL should be assessed in the game/using the game or outside of the game [41]. Bellotti et al. describe different types of assessment for students, including completion, in-process, and teacher assessments [38]. Completion assessment means finishing the activity or arriving at the correct answer [38]. In-process assessment deals with the decisions and step-wise processes the student considers during the game to arrive at the answer they selected [38]. Teacher assessment incorporates the educator’s observations about how the student performs throughout the activity [38]. These different forms of assessment can be used in combination to arrive at a more complete picture of true learning and to allow early feedback on all goals of the experience. Instructors can comment on incremental progress towards higher level objectives, while also incorporating both formative and summative assessment processes.

Another limitation with current assessment of GBL is that most studies measure knowledge using a pre-test and post-test. This methodology represents the most common type of assessment reported in the literature—likely due to ease of use—but possesses several drawbacks [38]. As Bellotti et al. notes, it is difficult to determine if post-test changes only represent learning in the GBL activity and not other outside factors, especially when students have many classes and interactions together [38]. In addition, Van Gaalen et al. mentions that many studies do not include adequately-defined control groups, which limits the quality of results [42]. As previously highlighted, typical post-test questions do not measure growth in sophisticated areas such as problem solving, critical thinking, and communication [38]. Vos et al. adds that environment and student characteristics unrelated to the game can sometimes influence assessment outcomes [43]. Furthermore, the performance of the instructor is important, as underprepared facilitators can inhibit smooth game integration, leading to decreased engagement and learning [43].

To address these concerns, some studies have considered the incorporation of assessments directly into the GBL activity and basing student performance on their actions in the game itself [38]. Completing assessments in the game reduces standardized testing that generally evaluates key concepts with multiple choice or other direct question types [38]. Transitioning assessments to the game keeps students engaged and allows for a more applicable form of direct and timely feedback for players [38]. Kim et al. describes these interventions as “stealth assessments” because students may not even realize they are being actively assessed in the engaging activity [39].

As an example, Vos et al. expects students will not only answer questions about the relevant topic, but also complete an active demonstration similar to what was completed in the GBL activity [43]. This dual level of accomplishment helps to establish that students achieved competency in both topic knowledge and practical application of the intended skills [43]. Competency is increased with multiple forms of assessments, such as formative assessments that incorporate direct and instant feedback [43]. Best practices also dictate that students should be taught at incremental levels throughout GBL, so that students may capture learning gains in a stepwise approach as the levels increase in difficulty [43].

## 6. Discussion

Game-based learning activities are designed to immerse students in interactive learning environments. Activities can vary greatly in features such as the amount of technology used in the exercise. The potential for great benefits and challenges exists with using large amounts of technology. Although more costly and likely to introduce logistical challenges, games that utilize advanced technology create more sophisticated and immersive experiences for users.

An immersive experience does not solely rely on technology, as several examples in the literature depict highly engaging experiences that utilize limited or no technology. Optimal GBL incorporates highly immersive features to maximize engagement; however, simple and direct games can also add value to the classroom and improve learning outcomes for students.

One common barrier associated with GBL is the number of resources needed to develop and implement a successful activity. Creating software with a third-party developer can cost thousands of dollars. In addition, other highly immersive experiences without major technology require significant resources in the form of time and personnel. This highlights the need for detailed publication of successes as well as failures to allow shared discovery and incremental progress in evaluating designs for effectiveness and practicality.

### 6.1. Future Steps

Assessment of GBL activities should be further considered and elucidated in the literature. Many diverse forms of assessment currently evaluate student performance, yet most published works focus on subjective outcomes that do not correlate well with advanced learning objectives. A defined assessment plan for GBL could provide consistency and guide educational researchers on best practices to evaluate activities. In addition, more research is needed on the feasibility and advantages of in-game versus out-of-game assessment. Optimized in-game assessment may provide benefit by evaluating more complicated problem-solving skills through active demonstrations. With these increased expectations, the authors also encourage the co-development of more sharing platforms and the expansion of formal publication opportunities for new GBL ideas. Broadly speaking, few attempts at GBL are published in the pharmacy education literature, and stricter assessment requirements could further increase the barrier for formal dissemination.

As courses continue to shift to more online instruction, educators create new models to interact and engage with students. Future research could address the modification and effectiveness of GBL in online or hybrid learning environments, especially for games with low technology integration.

### 6.2. Key Points and Takeaways

1. Game-based learning can provide an engaging environment for student learning.
2. Game-based learning is utilized and developed for a variety of pharmacy content areas.
3. Games with both advanced and minimal technology integration have the potential to improve educational outcomes.
4. Current assessment of game-based learning is limited and not well-defined, as most use pre- and post-tests. In the future, perhaps more in-game assessments combined with expanded outlets for new ideas may advance dissemination and improvements in student learning.

## 7. Conclusions

Many studies in the literature indicate that game-based learning may improve student engagement and knowledge. However, not all intended outcomes for GBL activities are captured by current assessment methods. GBL is applied to many disciplines and content areas throughout pharmacy education. Intentional design principles and technology increase immersion and enhance the student experience. There are many innovative examples of GBL in the literature that can be used as a springboard to integrate new activities in a variety of content areas.

## Figures and Tables

**Table 1:** Topics covered by GBL in the literature.

Content Areas Utilizing GBL
***Pharmacotherapy Focused***
Immunology
Diabetes Treatment Opioid Safety Cough Therapy Herbal Medicine Toxicology/Acute Care Pharmacotherapy Review
Therapeutic Decision Making
Medication Histories and Reconciliation
Nonprescription Pharmacy
***Not Pharmacotherapy Focused***
Drug Information and Literature Evaluation
Community Pharmacy Nonsterile Compounding Chemotherapy Good Manufacturing Practices
Leadership
Business of Healthcare
Healthcare Communication
Pharmacy Management

**Table 2:** GBL activities ranked by technology usage.

Technology Usage *	Design Features	References	Example Content Areas	Advantages/Disadvantages
High Tech/High Immersion	Advanced software simulations. Many 3D simulations. Some utilize player roles or avatars.	[14,15,16,17,18,26,27]	Community Pharmacy, Herbal Medicine, Immunology, Opioids	Advantage: High engagement, High quality experiences Disadvantage: Potential High cost, Time consuming to develop
Moderate Technology/High Immersion	Simulations followed by quiz tools. Fantasy League focused on investment. Mystery case tool that assigns patient characteristics for a case.	[11,13,28,29,30,31]	Health Care Industry, Medication Histories	Advantage: High engagement Disadvantage: Time consuming to develop
Moderate Technology/Low Immersion	Quiz format using online platforms/tools. Digital badges for completion of work.	[10,12,32,33]	Pharmacotherapy, Drug Information & Literature Evaluation	Advantage: Less cost and time consumption compared to high tech GBL activities Disadvantage: Less engaging than high tech/high immersion
Low Technology/High Immersion	Escape Room, Amazing Race^®^ design	[19,20,21,22,23,24,34,35]	Toxicology, Diabetes, Good Manufacturing Practices, Nonsterile Compounding, Pharmacy Leadership	Advantage: High engagement, Creative approach Disadvantage: Time consuming to develop
Low Technology/Low Immersion	Jeopardy^®^ questions, Name that drug	[36,37]	Review Style Games	Advantage: Ease of implementation in course, highly adaptable Disadvantage: Less engagement

* High Technology = Utilized advanced software or game design. Moderate Technology = Utilized basic software. Low Technology = Limited use of software.

**Table 3:** Design and Assessment of GBL.

Authors of Study	Content Area	Design	Summary of Results	Effectiveness
Khalafalla et al. [12]	Immunology–Transplantation	Divided students into teams for quizzes, cases, and Kahoot	Improved team-based scores on quizzes	Improved scores on quiz or post survey
Nabhani et al. [32]	Drug Information	Web-based quiz to assess retrieval ability in a national formulary.	93% of students felt the game helped them in the course. 55% of students had improved confidence. Significant improvement in quiz scores (*p* < 0.05)	
Kavanaugh et al. [20]	Treatment of Diabetes	Escape Room	Improved knowledge scores on post survey (*p* < 0.001)	
Berthod et al. [19]	Chemotherapy Good Manufacturing Practices	Escape Room	Increased scores on post survey (*p* < 0.001)	
Korenoski et al. [34]	Toxicology/Acute Care	Lock box kit stores clues to the game. Similar to escape room design.	Increased confidence related to toxicology and post-test scores	
Caldas et al. [21]	Nonsterile compounding	Escape Room	Increased median assessment scores (*p* < 0.001)	
Baker et al. [23]	Leadership Concepts	Escape Room	Significant increase in understanding of leadership concepts (*p* < 0.01)	
Eukel et al. [22]	Diabetes	Escape Room	Improvement in knowledge (*p* < 0.01)	
Richey Smith et al. [27]	Perspectives of patients in poverty	Online simulation using SPENT simulator tool	Improved post-survey scores (*p* < 0.001)	
Devraj et al. [11]	Pharmacy Management Course	Software App utilizing timed quizzes and multiple levels	Engaging app, but knowledge scores did not improve	Beneficial but no improvement in knowledge scores
Berger et al. [15]	Cough Therapy	Software that created 3D simulation of community pharmacy	No difference in clinical knowledge scores, however students felt more confident	
Bindoff et al. [14]	Community Pharmacy	Software game that created 3D simulation of community pharmacy	Students found the game enjoyable, but knowledge scores did not improve significantly	
Ee et al. [26]	Herbal Medicine	Mobile game utilizing simulations. Players manage a city specializing in herbal products.	No significant association between time spent playing the game and quiz scores (*p* = 0.236). Students felt they gained knowledge.	
Dicks et al. [37]	Nonprescription Pharmacy	Name that drug, Scavenger Hunts, Nonprescription Jeopardy	Examination scores did not improve in the GBL cohort of students. Improvements in course evaluations.	
Dell et al. [10]	Pharmacotherapy Review	Kahoot! to review key concepts	Student scores on the review game correlated with course grades. Students submitted questions used in the game.	Focused measurement on confidence/engagement/enjoyment/collaboration, leadership, communication
Abraham et al. [17]	Opioid Safety	Developed software allows players to participate with a character in multiple levels	Themes identified were avoidance of medication misuse and engaging game design	
Duffull et al. [28]	Therapeutic Decision Making	Patient simulation using software platform	Thematic analysis identified improvements of feeling in control and ability to make decisions	
Sando et al. [29]	Medication Histories and Reconciliation	Mystery case tool that randomly assigned patient characteristics for students to evaluate	Students felt the activity was valuable and applicable.	
Gorman [35]	Drug Information	Amazing Race challenges focused on using drug databases	Improved collaboration between instructors. More engaging for students	
Cusick [36]	Immunology Review	Jeopardy style game using clickers	Students found the game engaging	
Kayyali et al. [31]	Drug Information	Web-based software with quiz format and multiple player types to retrieve info from a national formulary	Most students would play the game again as a revision tool or because it was fun	
Wolf et al. [30]	Business of Healthcare	Fantasy League focused on investment	Increased confidence in topics surveyed	
Lam et al. [16]	Healthcare Communication	Software simulation including player avatars	Students found the software a worthwhile learning experience	
Cain [24]	Pharmacy Management	Escape Room	Escape room more engaging than traditional classroom experience	
Fajiculay et al. [33]	Drug Information and Literature Evaluation	Digital badges given to students for optional work	Increased confidence after obtaining a digital badge	
Barber et al. [18]	Immunology	Software simulation using different player roles	Choice impacted enjoyment in the student experience	
Fens et al. [13]	Community Pharmacy Patient Counseling and Prescription Processing	Software that simulates community pharmacy	Students value the game but want more direct feedback.	

## Data Availability

Not applicable.
